# Fabrication and Characterization of Nerolidol-Based Invasomes: Loading, Stability and Antimicrobial Applications

**DOI:** 10.3390/pharmaceutics18040410

**Published:** 2026-03-27

**Authors:** Gaetano Lamberti, Raffaella De Piano, Diego Caccavo, Sara Guarino, Lorenzo Bosio, Dante Greco, Clotilde Silvia Cabassi, Nicolò Mezzasalma, Costanza Spadini, Federico Righi, Marica Simoni, Susanna Bosi, Anna Angela Barba

**Affiliations:** 1 Dipartimento di Ingegneria Industriale, Università degli Studi di Salerno, Via Giovanni Paolo II n.132, 84084 Fisciano, SA, Italy; glamberti@unisa.it (G.L.); rdepiano@unisa.it (R.D.P.); dcaccavo@unisa.it (D.C.); 2 Dipartimento di Farmacia, Università degli Studi di Salerno, Via Giovanni Paolo II n.132, 84084 Fisciano, SA, Italy; sarguarino@unisa.it; 3 Dipartimento di R&S, Farmer SpA, Via Don D. Bertoldi, 63, 46047 Porto Mantovano, MN, Italy; lorenzo.bosio@farmer.it (L.B.); dante.greco@farmer.it (D.G.); 4 Department of Veterinary Science, University of Parma, Via del Taglio 10, 43126 Parma, PR, Italy; clotildesilvia.cabassi@unipr.it (C.S.C.); nicolo.mezzasalma@unipr.it (N.M.); costanza.spadini@unipr.it (C.S.); federico.righi@unipr.it (F.R.); marica.simoni@unipr.it (M.S.); 5 Centro Interdipartimentale di Microscopia Avanzata CIMA “Carlo e Dirce Callerio”, Dipartimento di Scienze Della Vita, Università degli Studi di Trieste, Via Fleming 31/B, 34127 Trieste, TS, Italy; sbosi@units.it

**Keywords:** nanoliposomes, invasomes, Nerolidol, simil-microfluidic technology, antimicrobic activity

## Abstract

**Background/Objectives**: Nerolidol (NER) is a sesquiterpene alcohol with recognized antimicrobial potential, whose applications as a pure substance are limited by hydrophobicity, instability, and cytotoxicity. Invasomes, i.e., liposomes with terpene ingredients, offer a strategy to improve their delivery; however, the NER loading limits compatible with vesicle integrity are still unclear. Here, Nerolidol-loaded invasomes were produced using a controlled simil-microfluidic coaxial injection process. **Methods and Results**: As a preliminary step, unloaded liposomes were fabricated to consolidate operating conditions and ensure their reproducible colloidal properties. Thereafter, formulations with progressively decreasing nominal NER loads were investigated to evaluate vesicle size, polydispersity, ζ-potential, encapsulation efficiency, effective loading, and stability. High nominal loads promoted turbidity, size increase (by agglomeration coalescence phenomena), and structural instability, whereas formulations containing approximately 1–2% NER achieved nearly complete encapsulation, Z-average ≈ 300 nm, |ζ| > 30 mV, and satisfactory physical stability. Antimicrobial and cytotoxic profiles of representative formulations, previously evaluated in an independent study are here reported only to contextualize the practical relevance of the optimized systems, while the present work primarily focuses on process–formulation aspects and loading/stability limitations. **Conclusions**: Overall, the present work identifies a realistic loading window for Nerolidol invasomes and highlights the suitability of the simil-microfluidic approach to obtain scalable, well-controlled formulations, providing a rational basis for their future biological assessment. Nerolidol invasome systems indeed can be considered a promising, versatile platform for antimicrobial applications, including prospective use in animal feed.

## 1. Introduction

Natural resources represent an invaluable reservoir of wealth for human needs; their respect and effective utilization are key challenges in promoting sustainable consumption and production models [1,2]. These models form the foundation of global political and economic strategies aimed at securing the well-being of both current and future generations (as outlined in Goals 3 and 12 of the United Nations Agenda 2030) [3,4]. To address these challenges, advanced technologies are required to transform natural resources into products with broad applications while continually intensifying process intensification paradigms (better process, responsible use of materials and energy sources, and reduced environmental impact) [5].

One of the most important areas for harnessing natural resources is the pharmaceutical and nutraceutical sectors, as evidenced by the millennia-long use of plants and herbs in traditional medicine [3]. Among the main limitations of using natural products are biodegradability and the safe dosing of compounds that can exert therapeutic/functional effects. For these reasons, the production of suitable delivery systems, as appropriate active/useful ingredients, is a key step in the development of new, effective, and safe pharmaceutical-nutraceutical products. Nanotechnology, by its various implementation forms, represents the most recent response to the need for preparing highly efficient dosage forms [6,7,8,9].

Drawing on previous experiences in producing nanoliposomal carriers via microfluidic nanotechnology [8,10,11,12], this study focused on developing lipid vesicles to encapsulate Nerolidol, a natural compound with powerful recognized healthcare features [13]. As widely shown in scientific literature, lipid vesicles, i.e., phospholipid-based colloidal particles featuring lipid bilayers enclosing a central aqueous or hydroalcoholic compartment [14,15], were purposely designed to leverage Nerolidol and other terpenes with significant antimicrobial, anti-inflammatory, antiparasitic, antifungal, antioxidant, and cancer-preventative properties [6,16,17,18,19,20]. Recent studies have further highlighted the relevance of terpene-based delivery systems and their interaction with biological environments [21,22].

Nerolidol is, in its natural form, also commonly used as a fragrant ingredient in the food industry (classified as a flavoring agent by the EU Food Improvement Agents and the Joint FAO/WHO Expert Committee on Food Additives—JECFA number 1646) and in cosmetic formulations such as fine fragrances, shampoos, toilet soaps, and other toiletries [18,23,24]. Moreover, Nerolidol in essential oils of plant derivatives is used also as feed additives (PFAs) in animal nutrition [25].

**Figure 1:**
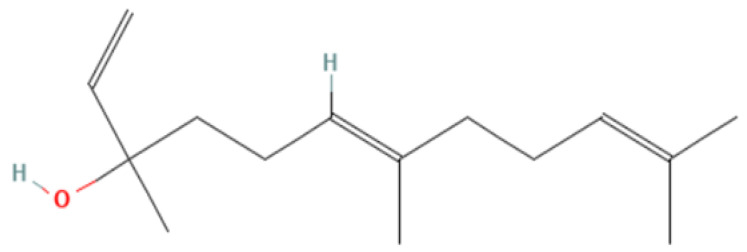
Nerolidol chemical structure: it features methyl groups at positions 3, 7, and 11, along with a hydroxyl group at position 3. Nerolidol exists as two geometric isomers: cis and trans, depending on the orientation around the central bond (https://pubchem.ncbi.nlm.nih.gov/compound/5284507#section=2D-Structure, accessed on 23 February 2026).

From a chemical standpoint, Nerolidol is a sesquiterpene alcohol and, biologically, a secondary metabolite widely found in plants adapted to diverse climatic conditions worldwide. Structure and main properties are reported in Figure 1 and Table 1, respectively.

Commonly, Nerolidol can be found in different species of lavender, tea tree, neroli, ginger, jasmine, and lemon grass, and as an abundant component in the essential oil (EO) of species such as *Cannabis sativa*, *Brassavola nodosa* and *Dalbergia parviflora* [13,26]. Nerolidol, therefore, can be obtained very easily, in particular from various parts of plants such as leaves, flowers, seeds, fruits, resins, twigs and wood. Based on literature references, leaves are the most common source for Nerolidol extraction. Seasonal variation is one of the main factors influencing the composition of essential oils in plants, including the concentration of Nerolidol [13].

Despite its biological activity, the direct use of Nerolidol is limited by its hydrophobic nature, low aqueous solubility, volatility, and potential cytotoxicity at elevated concentrations. Encapsulation in lipid-based carriers, such as liposomes, allows improved dispersion, enhanced stability, and controlled release, while also facilitating interaction with biological membranes. These features justify the use of liposomal systems over the direct application of the free compound.

Among the wide fields of scientific interest and applications, in this study, Nerolidol was selected to produce and test liposomal-loaded systems for its antimicrobial features. Many studies in the literature consider nanoliposomes (NLPs) as excellent vehicles for encapsulating antibacterial agents for different purposes: food and agriculture, cosmeceutics, and pharmaceutics [16,19,27,28,29]. Encapsulation overcomes the drawbacks of direct applications of antibacterial compounds, such as low stability and solubility; possible unwanted interactions with surrounding agents/ingredients, which allow possible side effects such as intolerances, decrease in antimicrobial efficiency, physical and chemical alterations (integrity, consistency, color, taste) [19,27]. Moreover, nanocarriers ensure, thanks to their high surface/volume ratio, an increase in solubility, dispersibility, and bioavailability while at the same time improving the controlled release of nanoencapsulated antimicrobial agents. Nanoliposomes, in particular, represent suitable vehicles due to their composition and structure: they are able to incorporate lipophilic, hydrophilic, and amphiphilic compounds, and their similarity to the external structure of bacterial cells leads them to more easily target bacteria [19]. Furthermore, the encapsulation of Nerolidol (and terpenes in general) in liposomal vesicles, whose formulation starts from the use of ethanol, also leads to an extraordinary potential of the resulting structures: an extreme flexibility of the liposomal architecture. The terminology “invasome” is used to indicate this kind of carrier, extraordinary vehicles, which are characterized by marked permeability throughout biological barriers, including animal mucosae and skin. For this reason, invasomes are particularly suitable for transdermal administration with all the innumerable advantages that this entails [30]. In many studies, it is highlighted the exploitation of terpenes in liposomes as drug permeability enhancers, especially in the formulation of transdermal drug delivery systems [28]. Indeed, terpenes are not only used for their direct functional properties (antibacterial, pharmacological, etc.) but also are specifically introduced into formulations to improve the therapeutic profile of other ingredients. In Table 2, in particular, the uses of Nerolidol as a permeability enhancer in drug control release are summarized.

In this work, we investigated the fabrication of Nerolidol-loaded invasomes by means of a controlled simil-microfluidic coaxial injection process. As a preliminary step, unloaded liposomes were produced and characterized to consolidate the operating conditions and formulation parameters that ensure reproducible vesicle formation and long colloidal stability. In parallel, the antimicrobial and cytotoxic properties of the obtained formulations were assessed in a previous study [33]. In the present work, these data are not introduced as novel results but are instead used to support the selection and interpretation of the most relevant formulations. Accordingly, the main objective of this study is not to provide new biological evidence but rather to investigate the process–formulation relationships governing Nerolidol loading, vesicle integrity, and stability. Building on previously validated operating conditions, we first explored high nominal Nerolidol loads to assess their impact on vesicle formation and colloidal stability. Based on these observations, the loading strategy was progressively refined to identify a realistic formulation window. The combined process–structure–stability analysis presented here not only provides quantitative insights into the actual loading limits of Nerolidol in invasomes but also demonstrates how controlled mixing conditions enabled by the simil-microfluidic approach can be exploited to define a realistic and reproducible formulation window and establishes a robust basis for subsequent biological applications. In this context, the novelty of the present work is not the use of terpene-based invasomes per se, which is already reported in literature, but the establishment of a process–formulation framework linking operating conditions, Nerolidol loading, and vesicle stability, enabled by the simil-microfluidic fabrication approach.

## 2. Materials and Methods

### 2.1. Materials

*Nerolidol and liposomal components*. Cholesterol (CHOL) (CAS no. 57-88-5), L-α-Phosphatidylcholine (PC) from soybean, type II-S, with a choline basis of 14–23% (CAS no. 8002-43-5), analytical grade ethanol (CAS no. 64-17-5), and Nerolidol (NER) (CAS no. 7212-44-4) were purchased from Sigma Aldrich (Milan, Italy) and used without further processing. Acetonitrile (CAS number: 75-05-8) for HPLC analytic control was supplied by Carlo Erba Reagents, DASIT Group Spa (Cornareto, Milan, Italy). Deionized water was produced using a laboratory deionizer.

Chemically, Nerolidol belongs to the class of sesquiterpenoids, a type of terpene comprising three consecutive isoprene units. As illustrated in Figure 1, its structure features methyl groups at positions 3, 7, and 11, along with a hydroxyl group at position 3. Nerolidol exists as two geometric isomers: cis and trans, depending on the orientation around the central bond. It exhibits solubility in ethanol and low solubility in water.

*Microbiological investigations*. For the verification of aseptic production conditions, culture media suitable for sterility controls were employed. In particular, Plate Count Agar (PCA) and Mueller–Hinton Broth (MHB) (purchased from Sigma Aldrich, Milan, Italy) were used as non-selective substrates to detect possible microbial contamination during the production and storage steps, in agreement with the sterile-production protocol optimized in this work. Sodium hypochlorite (NaOCl) and UV light were used for piping, containers and materials sanitization, with the aim of minimizing microbial burden while preserving vesicle integrity.

For the evaluation of the antimicrobial properties of Nerolidol-loaded invasomes, materials consistent with the reference study were adopted [33]. Mueller–Hinton Broth (MHB) and Mueller–Hinton Agar (MHA) were used for bacterial growth and quantitative assays, while a phosphate buffer solution (10 mM, pH 7.0) was employed for washing and standardization of the inocula. Antimicrobial tests were carried out on reference bacterial strains of veterinary relevance, selected because of their interest in potential applications in the animal-feed sector: *Staphylococcus aureus* (methicillin-resistant, ATCC 43300), *Escherichia coli* (ATCC 25922), *Salmonella enterica* serovar Typhimurium (ATCC 14028), *Enterococcus faecium* (ATCC 19434) and *Lactobacillus acidophilus* (ATCC 4356). These strains allow simultaneous assessment of both pathogenic bacteria and beneficial gut commensals, providing a biologically meaningful screening framework. All reference strains were purchased from ATCC^®^ Manassas, VA, USA.

### 2.2. Setup and Fluid-Dynamic Conditions for Invasomes Fabrication

#### 2.2.1. Setup and Fluid Dynamic Insights

**Figure 2:**
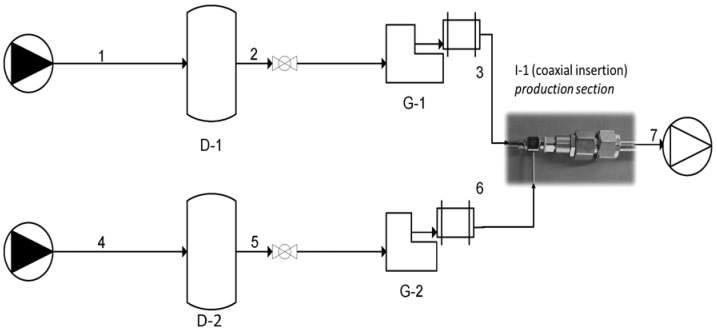
Simil-microfluidic piping schematization adapted from [11]. From upper left: (1–2–3) lipids/ethanol feed line; (4–5–6) water feed line; (D-1 and D-2) feed tanks; (G-1 and G-2) peristaltic pump-dampener groups; (I-1) coaxial flows insertion (particular to the used device: made in AISI 304 stainless steel shaft and in chrome and zinc-plated brass hub; size: Gauge 23—SKe Research Equipment); (7) water/ethanol vesicular suspension.

To overcome the challenges associated with traditional nanoliposome production, such as harsh operating conditions, use of toxic solvents, multiple post-processing steps, poorly controlled environments, low output volumes, and the high costs of microfluidic devices, the new technology, called ‘simil-microfluidic’, has been exploited. This technology provides precise fluid dynamics control and eliminates the mentioned limits, making it a promising solution for high-throughput nanovesicle manufacturing. It enables the production of homogeneous lipid vesicles in a single step at room temperature, directly at the nanoscale, by controlling the interaction of two phases through a coaxial insertion system where pumps deliver the lipid and the hydration solution. The fabricated simil-microfluidic apparatus was presented in detail in previous works [11,34]; an updated piping diagram representation is proposed in Figure 2.

For clarity, the main operating conditions of the simil-microfluidic process are summarized as follows: the aqueous phase (Vhs, deionized water) and the lipid phase (Vls, ethanol containing phospholipids, cholesterol, and Nerolidol) were injected in a coaxial configuration at a volumetric flow rate ratio of 10:1, corresponding to 7.5 × 10^−7^ m^3^/s and 0.75 × 10^−7^ m^3^/s, respectively. The process was conducted at room temperature.

Under the operative conditions tested and used in this study, a laminar regime is maintained for the resulting hydroalcoholic suspensions (N_Re_ = 240; and 8.25 × 10^−7^ m^3^/s mixing flow rate). At these conditions, the mixing between the two phases is predominantly driven by diffusion mechanisms [10]; the interdiffusion of water and the organic solvent phenomenon reduces lipid solubility, and this reduction, coupled with the thermodynamic instability of the phospholipid bilayer fragments’ edges, promotes curvature and closure of the bilayer fragments, leading to the formation of liposomal vesicles [35].

#### 2.2.2. Invasomes Fabrication 

The simil-microfluidic system is capable of continuous operation at high throughput by maintaining a constant ratio between the volumetric flow rates of the two phases. In this study, Nerolidol-loaded invasomes were produced using a 10:1 ratio of hydration solution volumetric flow rate (Vhs) to lipid solution volumetric flow rate (Vls). The operating conditions were set with a lecithin/cholesterol ratio of 5:1 *w*/*w* (theoretical PC:CHOL 2.35 g: 0.47 g in 50 mL, ethanolic phase, minimum used volume to each production batch) and a variable Nerolidol concentration. The load of Nerolidol was increased based on our previous research and literature evidence [11,34] exploring different theoretical loads from 1% up to 15% *w*/*w*.

The theoretical (nominal) loading of Nerolidol, defined as the mass fraction of Nerolidol relative to the total lipid phase (phospholipids + cholesterol + Nerolidol), is reported in Equation (1).

(1) $$Theoretical\;Load,\;\%=\frac{NER}{Total\;(PC+CHOL+NER)}\cdot 100$$

This definition is consistently used throughout the manuscript as the input formulation parameter.

Details on the prepared production batches are reported in Table 3. The unloaded first batch (Prod. 1, Unloaded Nanoliposomes, UN) was prepared as a reference system (control).

A second series of productions was then carried out, focusing solely on lower loads for reasons discussed in the following. Production at 1% was repeated, and a batch at 2% theoretical load was explored. Details on this second group of batches are reported in Table 4.

Overall, the invasome formulations consisted of L-α-phosphatidylcholine (soybean-derived) and cholesterol (PC:CHOL = 5:1 *w*/*w*), with ethanol as organic solvent (~9% *v*/*v* in the final mixture), and Nerolidol incorporated at theoretical loadings ranging from 1 to 15% (*w*/*w*) relative to the lipid phase.

### 2.3. Characterization Methods

#### 2.3.1. Separation Steps

After the fabrication procedure, to separate the pellet from the supernatant, a tangential flow filtration (TFF) step was performed. This process resulted in a retentate (concentrated pellet) and a permeate (supernatant or filtrate). The TFF membrane (Minimate^TM^ TFF Capsule 300KdOmega—PALL Laboratory, Port Washington, NY, USA) was installed in a custom-designed filtration loop equipped with two plungers moving in opposite directions. As one plunger fills, the other empties, ensuring continuous flow, as explained in [36].

As previously discussed in [11], tangential flow filtration was preferred over traditional ultracentrifugation due to its gentler impact on vesicle structures and its ability to minimize the risk of pellet resuspension in the supernatant. Centrifugation, moreover, causes a massive collection of all particulates, making it difficult to distinguish between individual particles or larger aggregates, such as those of NER in aqueous bulk, which can lead to inaccurate encapsulation yield determinations. Finally, tangential flow filtration was preferred over cross-flow filtration because the latter can quickly saturate the membrane and increase pressure, potentially causing vesicle rupture.

#### 2.3.2. Vesicles Size, Superficial Charge and Morphology Inspections 

The Z-average size, defined as the average hydrodynamic diameter, of the nanolipid vesicles was determined by dynamic light scattering (DLS). DLS measurements were performed under room conditions after diluting (with distilled water in ratios ranging from 1:3 to 1:4) and sonicating the sample suspensions. Each sample was measured at least in triplicate, and results are reported as mean ± standard deviation.

Along with the Z-average size, measurements of the zeta potential were performed. Zeta potential is the primary force governing the interactions (repulsion/attraction) between particles and is highly sensitive to the composition of the species in the dispersion. A high zeta potential value (typically > |30| mV) ensures that suspended particles are well-dispersed, reducing the probability of agglomeration and making the solution stable over time.

Macroscopic evaluations of the samples, assessing transparency and the presence of precipitates, were performed through visual inspection and subsequently quantified by a turbidimetric method (PCE-TUM 20 turbidimeter, supplied by PCE ITALIA srl, Capannori, Lucca, Italy).

Morphological characterization of vesicle structure was performed by transmission electron microscopy (TEM), allowing identification of both well-defined vesicles and non-vesicular structures. TEM images were acquired using a Philips EM 208 instrument, fitted with an Olympus Quemesa camera (EMSIS GmbH, Münster, Germany) and RADIUS software. Approximately 10 μL of the washed pellet sample was diluted with distilled water and placed onto a carbon-coated copper specimen grid (200 mesh, Electron Microscopy Sciences). Finally, the sample was negatively stained with a 1% (*w*/*v*) uranyl acetate solution.

#### 2.3.3. Encapsulation Efficiency and Effective Load 

Encapsulation efficiency (EE) is defined as the ratio of the amount of Nerolidol encapsulated within the liposomes to the total amount of Nerolidol used. 

EE is calculated using Equation (2):

(2) $$EE,\;\%=\;\frac{Encapsulated\;NER}{Total\;NER}\cdot 100$$

Effective load (EL) is defined as the amount of Nerolidol encapsulated within liposomes relative to the total mass of lipids and Nerolidol. 

Effective Load is calculated using Equation (3):

(3) $$Effective\;Load,\%=\;\frac{Encapsulated\;NER}{Total\;(PC+CHOL+NER)}\cdot 100$$

To assay entrapped Nerolidol in liposomal vesicles and in supernatants phase, High-Performance Liquid Chromatography (HPLC) technique was used (Agilent Technologies 1260 Infinity instrumentation, Agilent Technologies Italia SpA Cernusco sul Naviglio Milan, Italy). At first, a calibration curve was defined for the analyte of interest in an appropriate solvent (ethanol) using solutions of known concentration (calibration standards). The applied analytical method was derived from similar analytical protocols reported in scientific literature [37,38]. In detail, an isocratic mixture of acetonitrile: water (60:40) was used as the mobile phase, and the commercial Kinetex^®^ 2.6 µm XB-C18 100 Å column was used as the stationary phase. For the determination of Nerolidol, a wavelength of 210 nm was used due to its maximum absorption wavelength. The calibration curve obtained by using standard solutions gave a linear fitting (y = 34.709x, with x as NER concentration, and a Pearson coefficient R^2^ of 0.9998). The samples subjected to chromatographic analysis were, when necessary, diluted and/or sonicated.

All measurements were performed in triplicate, and results are reported as mean ± standard deviation.

#### 2.3.4. Short- and Long-Term Stability

To evaluate both short-term (45 days) and long-term (6 months) stability, different batches of produced invasomes were stored in airtight containers under refrigerated conditions (4 °C), as reported in literature studies [14,18,20].

#### 2.3.5. Sterile Productions Control and Antimicrobial Assays 

To evaluate the antimicrobial activity of liposomal Nerolidol, sterile formulations were developed. After identifying the formulations of greatest interest (1% Nerolidol loading), a preparatory procedure was established through iterative testing.

Literature studies have widely highlighted that for obtaining sterile liposomal systems, the best production strategy is aseptic production rather than the application of terminal processes. This is because the presence of sterilizing agents, whether physical or chemical, can compromise the stability of vesicles due to phenomena of oxidative degradation of lipids or bilayer disruptions by the formation of free radicals [39,40,41]. Thus, the ethanolic phase was prepared in a controlled environment (laminar flow hood with UV light exposure), and sterile water was used as the aqueous phase. The bench-scale simil-microfluidic setup was positioned within a laminar flow hood equipped with a UV lamp to sterilize the work area and prevent cross-contamination. All containers used (for storing feed phases and production batches) were washed with a 3.8% sodium hypochlorite solution followed by sterile water rinsing. Finally, the production plant (piping) underwent sodium hypochlorite flushes (with overnight exposure to the oxidizing agent) and was subsequently rinsed with sterile water.

*Sterile production control*. Microbiological controls were performed using standard procedures for the detection of contaminants with non-selective culture media. In detail, 250 μL aliquots of each sample were inoculated onto Plate Count Agar (PCA) and incubated at two different temperatures: 25 °C and 37 °C. Additionally, 1 mL aliquots of the same samples were incubated in 9 mL of Mueller–Hinton Broth (MHB) at 37 °C for 24 h. Subsequently, 250 μL of the resulting broth was plated onto PCA and incubated at 25 °C and 37 °C. The results, indicating microbial growth, were recorded as a positive or negative observation (qualitative assessment) of colony formation on the third day post-incubation.

*Antimicrobial assays.* The antimicrobial activity of the formulations against reference bacterial strains was previously assessed using a Time–Kill assay at different contact times, as reported by Mezzasalma et al. [33]. In the mentioned study, all antimicrobial evaluations were performed following the Clinical Laboratory Standard Institute Method [42]. Specifically, all reference strains were cultured in Mueller–Hinton Broth at 37 °C under aerobic conditions for 24 h, except for the *E. faecium* and *L. acidophilus*, which were incubated in microaerophilic conditions. Subsequently, bacterial suspensions were centrifuged and resuspended in phosphate buffer; the suspensions were then standardized spectrophotometrically (OD600 = 0.08–0.13) to obtain approximately 10^8^ CFU/mL and subsequently diluted to reach a final inoculum of 5 × 10^5^ CFU/mL in the assays.

Time–kill assays were conducted by incubating bacterial suspensions with free nerolidol, Unloaded Nanoliposomes (UN), or Nerolidol-loaded invasomes (denoted as Loaded Nanoliposomes, LN) at various concentrations. Free nerolidol was tested at concentrations ranging from 4000 to 7.81 μg/mL. Nerolidol-loaded invasomes were evaluated at concentrations ranging from 2500 to 9.77 μg/mL of total formulation, corresponding to nerolidol concentrations from 25 to 0.097 μg/mL. Unloaded Nanoliposomes were tested at the same concentration range as the loaded formulations (2500 to 9.77 μg/mL).

For each reference strain, bacterial growth was assessed after 2, 4, 6, and 24 h of incubation at 37 °C under aerobic or microaerophilic conditions, depending on the bacterial species. At each time point, 10 μL aliquots were plated onto Mueller–Hinton agar (MHA), and the plates were incubated under appropriate conditions. Following incubation, colony-forming units (CFUs) were counted for each concentration and time point.

All measurements were performed in triplicate, and results are reported as mean ± standard deviation. Growth controls and sterility controls were included in every experimental set.

Time–kill experiments were performed by incubating the bacterial suspensions with free Nerolidol, Unloaded Nanoliposomes, or Nerolidol-loaded invasomes at different concentrations for Free-Nerolidol (from 4000 to 7.81 μg/mL), Nerolidol-loaded invasomes (from 2500 to 9.77 μg, containing from 25 to 0.097 μg/mL of Nerolidol), and Unloaded Nanoliposome (from 2500 to 9.77 μg). For each reference strain, bacterial growth was quantified after 2, 4, 6 and 24 h of incubation at 37 °C in aerobic/microaerophilic conditions based on the bacteria. Ten microliters were plated on Mueller–Hinton Agar (MHA), and the plates were incubated. After incubation, for each experimental point and tested concentration, colonies were counted. For each assay, three experiments, each comprised of three replicates, were performed, including growth (GC) and sterility controls.

### 2.4. Statistical Analysis

Statistical analysis was performed to evaluate the effect of storage on the physicochemical properties of invasomes at 1% and 2% Nerolidol loading. In particular, Z-average, polydispersity index (PDI), zeta potential, and effective loading were considered. For each parameter, data obtained from fresh and aged samples were normalized by dividing each value by its corresponding initial (fresh) value in order to allow direct comparison of relative variations over time. Differences between fresh and aged samples were assessed using a two-tailed paired Student’s *t*-test, as the same formulations were analyzed before and after storage. Statistical analyses were carried out using Microsoft Excel. A *p*-value < 0.05 was considered statistically significant.

## 3. Results and Discussion

### 3.1. Fabrication of Unloaded Nanoliposomes: Effect of Constituents

As introduced in the Section 2.2.2 Invasomes Fabrications, an initial production was carried out on Unloaded Nanoliposomes (UN in Table 5) with the dual purpose of checking the reproducibility of vesicular production by the simil-microfluidic apparatus and to establish a reference system for comparison with liposomal suspensions containing a terpene load.

Achieved Unloaded Nanoliposomes have shown the following characteristics: Z-average [nm] (305.8 ± 24.2), PDI (0.33 ± 0.028), and Zeta Potential [mV] (−41.3 ± 12.1) (Table 5). The results obtained are consistent with the experimental results of previous research using the same apparatus and operational conditions, confirming the reproducibility of liposomal suspensions produced with the developed methodology: [9,12] (Z-average 260 ± 1.0 nm, PDI 0.40 ± 0.01 and Zeta potential −40 ± 1 mV), [8] (Z-Average 252.8 ± 2.1 nm, PDI 0.38 ± 0.02 and Zeta potential −35.4 ± 0.8 mV), [34] (Z-Average 246.3 ± 1.10 nm, PDI 0.37 ± 0.04 and Zeta potential −35.2 ± 0.83 mV).

The variation in Zeta potential and vesicle size is likely attributed to the soy lecithin (food grade E322), which consists of a blend of phospholipids along with triglycerides, fatty acids, and carbohydrates. The phosphatidylcholine (PC) content fluctuates across production batches, typically ranging from 18–25%, and this variability can influence both the size and Zeta potential of the liposomal vesicles.

The formulation used for the liposomal production is based on previous research work, as mentioned before. Specifically, following the prior work, the decision was made to incorporate cholesterol into the recipe. Cholesterol is a steroid with a rigid structure and amphiphilic characteristics. It has a hydrophilic, polar part composed of hydroxyl groups and a hydrophobic, non-polar part made up of the tetracyclic ring system and a flexible side chain. Cholesterol is located near the polar groups but also fits within the membrane, where it interacts with the phospholipid acyl tails, limiting their movement.

This accommodation of cholesterol into the membrane affects various membrane properties, including fluidity, permeability, elasticity, lipid transition temperature, phospholipid packing, and plasma stability [17].

It has been observed that the effect of cholesterol on membrane rigidity (by increasing the microviscosity of the lipid membrane) and vesicle stability during storage is concentration-dependent [43]. At cholesterol concentrations lower than 10% *w*/*w* relative to membrane phospholipids (PC: CHOL 5:0.5), added cholesterol does not significantly increase membrane rigidity. However, at higher concentrations, an increase in rigidity is observed [17]. In this study, the PC: CHOL ratio was set to 5:1 (17% *w*/*w*).

Ethanol, unlike cholesterol, preferentially accommodates near the polar head of phospholipids, causing disruptions in their polar domain. Furthermore, its potential presence in the phospholipid bilayer leads to the formation of zones with micropolarity, resulting in increased hydration of the membrane. These effects lead to an increased exposure of phospholipids to oxidation and hydrolysis processes, making the vesicles more deformable and less stable. The characteristic flexibility (deformability and elasticity) of the vesicles makes them suitable for use in transdermal delivery. However, this flexibility simultaneously reduces their structural stability, leading to aggregation and the formation of giant lamellar structures, and decreases their shelf life over time, even due to mild thermal effects. Ethanol also contributes to the determination of the Zeta potential of lipid membranes [17].

**Figure 3:**
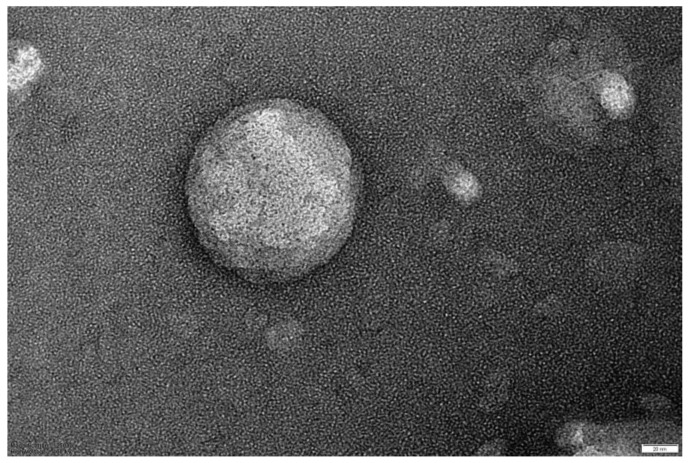
TEM image of unloaded liposomal vesicles (bar 20 nm).

In this work, summarizing, nanometric unloaded vesicles are achieved, with a negative surface charge, mainly attributed to the phospholipid nature of the membrane constituent (PC), which has phosphate groups at the end of the chain rather than the ethanol effect (due to its low content, 9% *v*/*v*). Morphology of vesicles, observed by TEM investigation (Figure 3), appears spherical with a fair smooth structure, confirming a complete self-assembly of the basic constituents (PC, CHOL).

### 3.2. Invasomes Production 

#### 3.2.1. Effects of Theoretical High Load

**Figure 4:**
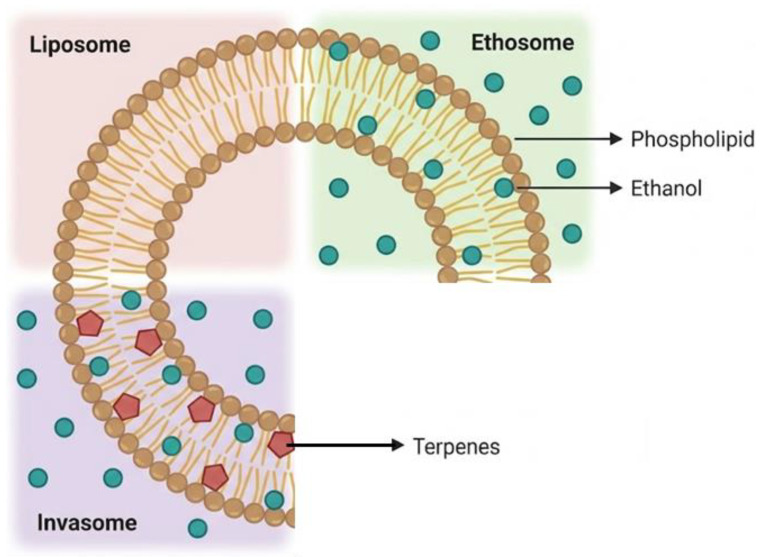
Denominations of liposomal vesicles based on their composition (redrawn from Emanet et al. [45]).

As explained in the introduction, the production of liposomal vesicles with terpenes, in the presence of ethanol, leads to the formation of delivery systems called invasomes [6,15,17,18,32]. More precisely, as sketched in Figure 4, depending on the presence or absence of terpenes and the ethanol concentration (as % *w*/*v* or % *v*/*v*), different terms are used for the liposomal-based vesicles: invasomes (3% *w*/*v*—[20]; 4% *v*/*v*—[44]; 10% *v*/*v*—[14]) and ethosomes (up to 50% *w*/*w* ethanol or other volatile alcohol). The scientific literature reports various formulations and technical processes to fabricate invasomes [15,17,18,20]. In particular, in addition to the different membrane lipids and the presence of ethanol, formulation differences include cholesterol presence [17,20], or cholesterol absence [28]; terpenes as active ingredients [20]; and terpenes and other active ingredients (terpenes are added for their enhancer function) [18]. The main objective of the mentioned research has been the preparation of more efficient drug delivery systems for the topical delivery of active molecules (invasomes are more flexible structures capable of penetrating the stratum corneum compared to classical liposomes) or added substances for beverages, food, or packaging materials to exploit the antimicrobial properties of terpenes [26,27].

In this work, ethanol and water are mixed in a 1:10 ratio, which corresponds to a 9% *v*/*v* ethanol concentration. Regarding the lipidic and terpene composition, different batches are considered, with the only difference being the theoretical load of Nerolidol (the lipid composition is the same for all the batches prepared), as reported in Table 3. The objective was to investigate the impact of Nerolidol load on liposomal architectures fabricated by the simil-microfluidic techniques, aiming to achieve the highest load in stable structures. The goal was to assess how the composition of Nerolidol affected the system while keeping the base ingredient ratio (PC: CHOL 5:1) constant. Based on existing scientific literature (as previously mentioned, Nerolidol is utilized as a functional agent and enhancer), a Nerolidol load range of 1% to 15% was selected. This range aligns with typical loads used to harness its enhancer properties [15,18,28] or for its antimicrobial effects [26] and provides a basis for comparison with similar preparation methods and lipid compositions in liposomal systems containing functional lipophilic molecules [8,34].

Thus, immediately after preparation, for all the prepared batches, qualitative and quantitative observations were made. Batches with higher Nerolidol loads showed the presence of aggregates and precipitates, as well as uneven colors, as the terpene content increased Table 5. DLS and turbidimetry results (Table 5) confirm a marked increase in vesicle size, polydispersity, and turbidity with increasing NER loading, indicating progressive structural destabilization. Dimensional and turbidity data (Table 5) indeed highlight that both the dimensions of the vesicles and the polydispersity index increase with the theoretical load of Nerolidol, in line with observed and measured turbidity. These results were consistent, at first glance, with the scientific literature (even though they refer to lower loads than 15% [15,17]): invasome dimensions increase with the load of terpenes, and the morphology, typically spherical, becomes oval and eventually leads to malformed vesicles.

Moreover, all batches prepared with variable loads were examined via HPLC to determine effective load and encapsulation efficiency. Results are reported in Table 6.

At this stage, it is important to distinguish between theoretical load, effective load, and encapsulation efficiency. The theoretical load represents the nominal fraction of Nerolidol introduced in the formulation, while the effective load reflects the actual amount associated with vesicular structures after separation. Encapsulation efficiency, instead, expresses the fraction of Nerolidol not detected in the supernatant and may therefore be overestimated in the presence of aggregates or phase-separated terpene. As a consequence, under high-loading conditions, high apparent encapsulation efficiency values (Table 6) do not necessarily indicate successful vesicle incorporation but may result from sequestration phenomena and non-vesicular partitioning of Nerolidol.

The observed instability at high Nerolidol loading can be attributed to its insertion within the phospholipid bilayer, where it perturbs lipid packing and increases membrane fluidity. At low concentrations, Nerolidol is accommodated within the bilayer with limited structural disruption; however, at higher loadings, the reduced lipid/terpene ratio leads to excessive bilayer disorder, promoting the formation of aggregates and non-vesicular structures. Indeed, the action of the included molecules, specifically the ability of Nerolidol to interact with the phospholipid bilayer due to its hydrophobic nature (log P_NER_: 5 in the BASF technical sheet, 5.32 in [30]; 5.68 in [13]), and the increasing concentration, led to the speculation that formulation and thermodynamic factors, related to the prepared batches, do not contribute to the formation of stable vesicles. High loads result in the dispersion of lipid fragments (rupture or vesicle malformation) in the hydroalcoholic bulk (with a polar character), causing terpene sequestration, not encapsulated. This explains the misleading data of encapsulation efficiency (see Table 6).

**Figure 5:**
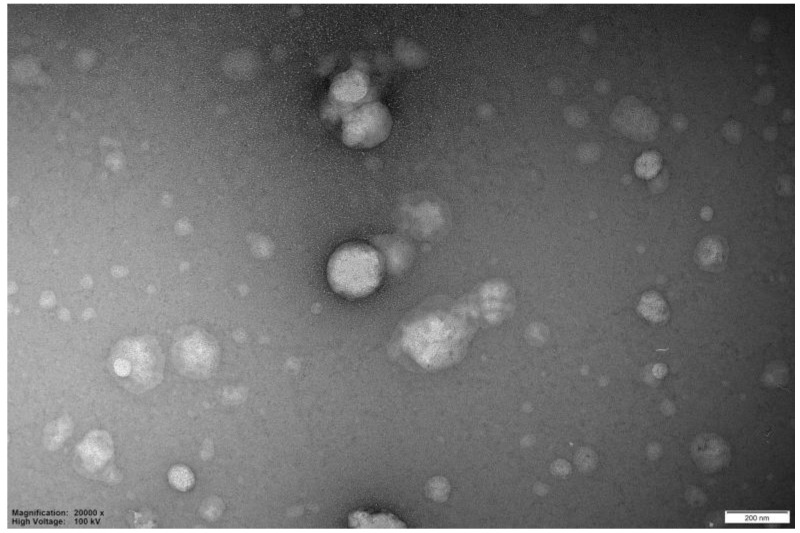
TEM image of batch INV. NER 15% (bar 200 nm).

**Figure 6:**
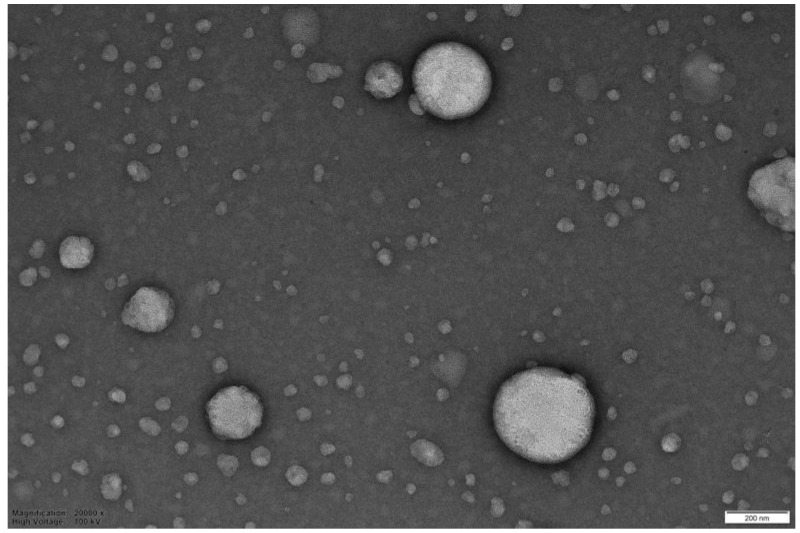
TEM image of batch INV. NER 15% after removal of larger aggregates (bar 200 nm).

The TEM investigations, conducted specifically to explore the morphology of the dispersed particulate matter, confirmed the presence of both aggregated systems of undefined shape and varying sizes, as well as structures with a rounded morphology typical of lipid vesicles. The following figures show TEM images related to the observation of samples with a theoretical load of 15% Nerolidol (Figure 5 and Figure 6). As can be inferred from the images, the electron microscopy examination confirms the results of turbidimetry and DLS investigations, highlighting the presence of many structures classifiable as lipid fragments and aggregates of varying sizes (Figure 5) and well-formed liposomal vesicles in Figure 6. This latter image is captured after tangential filtration of the sample to remove larger aggregates.

Due to its lipophilic nature (but also the possession of local amphiphilic properties attributed to the -OH group [18]), Nerolidol interacted with the lipids used in a more or less stable manner (as will be discussed further) according to a sort of “threshold” ratio. While the lipid-terpene interaction can be considered regardless of whether the bilayer-organized fragments close into vesicles, the formation and stability of the latter depend on the terpene load.

At low Nerolidol loads, a higher lipid/terpene ratio favors the interaction of Nerolidol with the hydrophobic tails of the phosphatidylcholine (noting that cholesterol also competes for these acyl chains). Subsequently, with the interdiffusion of water into the ethanolic phase (the medium containing both Nerolidol and lipids), lipid solubility decreases. This reduction, combined with the thermodynamic instability of the edges of the bilayer fragments, induces curvature and the closure of the fragments to form vesicles. Nerolidol thus remains incorporated within the lipid bilayer; due to its chemical and structural nature, it orients and accommodates itself parallel to the lipid lamellae, reducing their compactness [18]. Despite exerting a lipid rearrangement effect (spacer activity), the overall architecture of the vesicles remains stable.

Conversely, at higher Nerolidol loads, the lipid/terpene ratio decreases, leading to greater competition for interaction with the hydrophobic tails. The increased polar character of the microenvironment surrounding the bilayers—driven by water interdiffusion—promotes the formation of anomalous vesicles or aggregates. These structures represent the thermodynamic stabilization response of the highly heterogeneous lipid/lipophilic formations created by the sequestration of Nerolidol, equivalent to the process that leads to fragment closure at low loads. However, unlike standard vesicular formations, these structures are less stable due to both the higher terpene content and their physical conformation, resulting in continuous structural breakdown and the subsequent formation of precipitates. This hypothesis stems from observations conducted on batches stored for 6 months at 4 °C [18,19,20,44]. In the presence of well-formed vesicles, no statistically significant changes in size or PDI were expected. In fact, new DLS measurements on production batches showed a significant variation in mean size values only for those lots prepared with a high quantity of Nerolidol, as shown in the following Table 7.

The Z-average values for the batches with a load higher than 5% all significantly decreased, with a notable increase in size heterogeneity (high SD values; higher polydispersity), clear signs of structural reorganization, possibly due to fragmentation, of the larger aggregates produced.

Ethanol, also present in the formulations used in all production batches, contributes to the structural breakdown effects by fluidizing the lipid layers, as widely reported in the literature [17] and mentioned in the previous paragraphs. However, its action should always be considered in relation to the type of terpene used and its concentration. The concentration used in this study (1:10 ethanol: water, 9% *v*/*v*) is considered reasonably less significant (considering the stability of the liposomes without load) compared to that exerted by Nerolidol, especially for high-load preparations. In ethosomes, for example, the presence of high ethanol (or other volatile alcohols) concentrations (<50% *w*/*w*, optimal values between 20 and 30% *w*/*w*), in the absence of terpenes, provides the lipid structure with stability and, above all, extraordinary elasticity, making them highly effective for transdermal delivery [14,15]. The maintaining stability of vesicles is due to a modification of the net surface charge (although in many cases other ingredients are added for stabilization purposes); but over 45% the excessive permeability of the membrane causes its destabilization [46].

Considering the results obtained, which confirm findings in the most recent scientific literature, it can be concluded that the fluidizing effect of Nerolidol on phospholipid bilayers does not allow the production of invasomes with very high effective loads. The presence of cholesterol or the different nature of the phospholipid, while playing a role in the stability of the vesicles, does not mitigate its fluidizing action.

It is possible to conclude that Nerolidol loads above ~2% (*w*/*w*) lead to structural destabilization, aggregate formation, and misleading encapsulation metrics, making them unsuitable for stable invasome formulations.

#### 3.2.2. Invasomes with Suitable Load 

Following the experimentation with higher Nerolidol loads, research activities shifted focus to the production with lower Nerolidol loads (1% and 2%), defined as effective load productions. “Effective load production” means that Nerolidol is successfully encapsulated, stable, and potentially usable for therapeutic and antimicrobial activities. The characteristics (size, PDI-Z potential, PDI-effective load, encapsulation, efficiency) of fresh and aged batches are reported in Table 8.

The fresh batches displayed homogeneous appearances. The turbidimetric analysis was consistent with low-load previous productions, and the dimensions had an average of 300 nm with a moderately polydisperse distribution. Zeta potential analysis confirmed a very good stability, and, finally, effective loads and encapsulation efficiency showed a high-performance process. Aged products, as reported in Table 8, showed a slight increase in size and dimensional inhomogeneity, a low variation in Nerolidol content for the batch of 1%, and a more pronounced decrease in Nerolidol for the 2% lot. In conclusion, these productions of invasomes can be considered globally stable, in agreement with literature data. Although the studies refer to different terpenes (and lipid compositions), they all agree on the production of stable systems with good to excellent encapsulation efficiencies, but only for low effective loads (below 2%).

It is then possible to conclude that Nerolidol loads in the range of 1–2% (*w*/*w*) ensure a favorable balance between encapsulation efficiency, vesicle integrity, and colloidal stability, representing a realistic formulation window. From an application perspective, it should be noted that the direct use of Nerolidol at high concentrations may raise cytotoxicity concerns. In this context, encapsulation within invasomes and the identification of a limited loading window (≈1–2% *w*/*w*) are particularly relevant, as they enable controlled delivery while avoiding excessive terpene exposure.

Compared to conventional liposomes, invasomes are characterized by increased membrane flexibility due to the presence of terpenes and ethanol, which enhances their interaction with biological barriers. In contrast, nanoemulsion systems, while effective for solubilizing lipophilic compounds, lack the bilayer structure and controlled encapsulation capability typical of lipid vesicles. In this context, the simil-microfluidic approach adopted in this work provides an additional advantage by enabling controlled, continuous production and improved reproducibility compared to conventional batch preparation methods.

#### 3.2.3. Fabrication Sterility Control 

As previously reported, liposomes are structurally fragile and sensitive to standard operational processes. Conventional terminal sterilization (heat, radiation, chemicals) often causes lipid degradation, cargo leakage, and changes in particle size or charge. To preserve their integrity, aseptic manufacturing is the most reliable alternative, ensuring sterility through controlled environments throughout the entire production cycle [39,40,41,47].

To simulate an aseptic environment during the production of liposomal Nerolidol, controls on batches (1% in Nerolidol) were performed with the aim of observing any microbiological activity (by PCA and MHB described incubation procedures). The results showed the absence of microbial growth, confirming the effectiveness of the sodium hypochlorite solution in the cleaning of pipes and production environments and of UV treatment of ingredients.

Moreover, the produced invasomes have been once again characterized in order to observe any impact of UV exposure on the vesicular structures (Z Average, loading, encapsulation efficiency) [41]. Upon visual examination, the suspensions appeared free of precipitates, with the very slight opalescent coloration typical of all the productions carried out. Sterile productions exhibited vesicles with comparable size and characteristics to those produced without UV irradiation. In detail, the vesicles exhibited a size average of 307 nm, a PDI of 0.34, a 1% loading, and 99.41% encapsulation efficiency. Thus, although the measurements refer to macroscopic characteristics, no alterations due to UV exposure are discernible. It is reasonable to assume that the limited exposure times (20 min) and the diluted environment (hydroalcoholic bulk) do not allow the establishment of degradative processes such as, for example, lipid peroxidation (oxidative degradation of lipids) or an increase in the permeability character of the lipid bilayer due to the formation of free radicals [40]. This kind of product was used to perform the antimicrobial assays.

#### 3.2.4. Antimicrobial Assays 

It is important to note that the antimicrobial data discussed in this section were previously published [33] and are not presented here as novel findings. Their inclusion is intended to support the interpretation of the optimized formulations identified through the present process–formulation study. The antimicrobial activity of the nanoliposome formulation, prepared according to Section 3.1, i.e., without Nerolidol, designated as Unloaded Nanoliposomes (UN), and those obtained as described in Section 3.2, containing Nerolidol, referred to as Loaded Nanoliposomes (LN, or liposomal Nerolidol), was investigated by Mezzasalma et al. [33]. In both cases, the stock suspensions had a lipid concentration of 5 g/L, corresponding to 5000 μg/mL. In LN, Nerolidol represented approximately 1% (*w*/*w*) of the lipid content, yielding a Nerolidol concentration of 50 μg/mL. In the cited study, these suspensions were subjected to sequential 1:1 dilution prior to antimicrobial testing. The first dilution step, therefore, resulted in suspensions containing 2500 μg/mL of lipids and 25 μg/mL of Nerolidol, indicated using the shorthand notation 2500/25.0. Subsequent dilutions (e.g., 1250/12.5, 625/6.25, etc.) retained this notation, which simultaneously reports lipid and Nerolidol concentrations (μg/mL) for LN, while indicating only lipid concentration for UN. In the same work, both liposomal suspensions (at different dilutions) and free Nerolidol solutions were tested against five bacterial strains of veterinary relevance, and viable counts were determined as CFU/mL over time. The results are reported in the Supplementary Tables (S1–S8) in Mezzasalma et al., 2025 [33], whereas in the present work, the data of the time-to-kill assay are expressed as “average inhibition” versus time. Average inhibition is defined as $AI=\left(1-CFU\left(treat\right)/CFU\left(control\right)\right)\times 100$; because the Supplementary data are reported as ${log}_{10}\left(CFU/mL\right)$, the CFU values must first be back-calculated.

**Figure 7:**
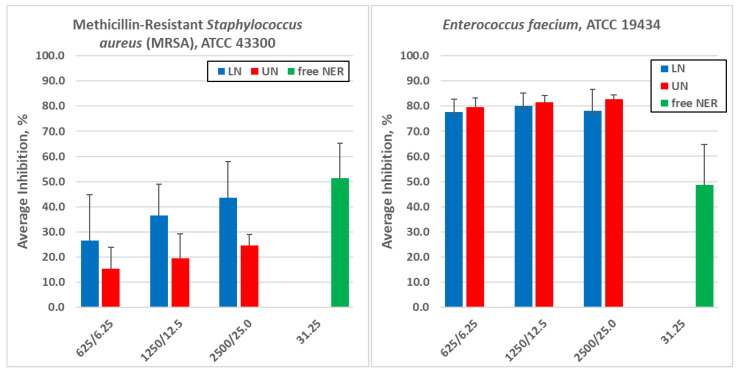

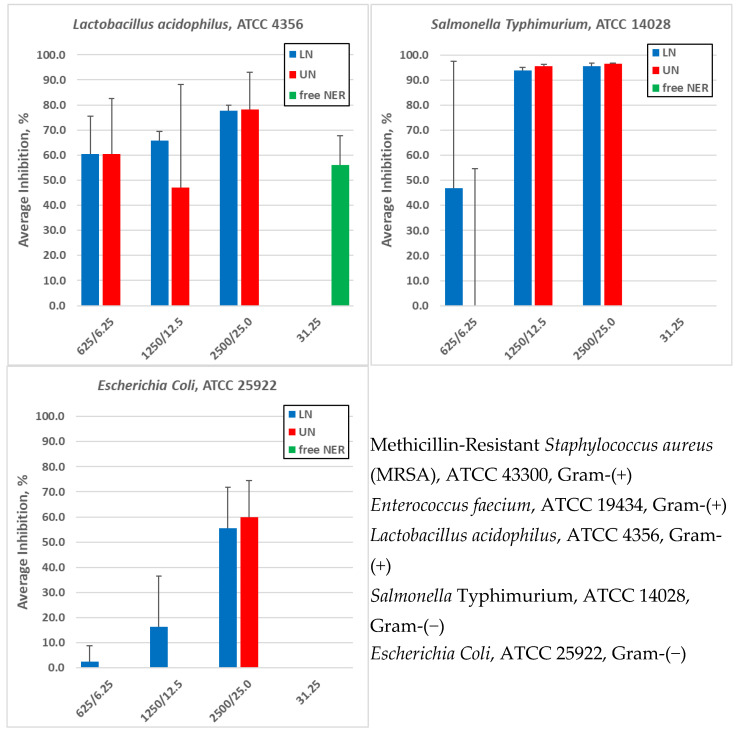
Summary of antimicrobial effects of free Nerolidol (NER), Unloaded Nanoliposomes (UN) and Nerolidol-loaded invasomes (LN) against five bacterial strains of veterinary interest. Graphs were built based on calculations performed on the data reported in the Supplementary tables in [33], considering the average inhibition at 2 h for selected concentrations (free NER: 31.25 μg/mL; UN and LN: 2500/25.0, 1250/12.5 and 625/6.25). The figure highlights the onset of measurable CFU reduction for each treatment and microorganism, allowing comparison between the effects attributable to Nerolidol and those associated with the liposomal matrix alone.

For the purpose of the present work, those datasets were considered, for each strain and treatment, the inhibition observed at 2 h [33]. The data considered interesting for this study were synthesized in Figure 7 and Table 9. Specifically, for free Nerolidol, the concentration of 31.25 μg/mL was considered, as this represents the lowest level at which measurable effects could be detected on the three Gram-positive strains, while no appreciable inhibition was observed on Gram-negative strains at any dose. For UN and LN, the analysis focused on the three highest concentrations (2500/25.0, 1250/12.5 and 625/6.25), which were those consistently associated with measurable effects across all strains. A first clear observation is that free Nerolidol exhibits activity primarily against Gram-positive bacteria, where measurable inhibition appears already at relatively low concentrations. However, this effect is not observed against Gram-negative species, confirming the protective role of the outer membrane in these organisms.

The behavior of Unloaded Nanoliposomes is particularly instructive. Although devoid of Nerolidol, UN still produces detectable inhibition at the highest lipid concentrations and, in some cases, in a slightly dose-dependent fashion. This confirms that part of the antimicrobial response may derive from physical interactions between lipid vesicles and bacterial envelopes rather than from the active compound itself. Importantly, when lipid concentration decreases, this effect is attenuated or disappears, indicating that it is a concentration-driven phenomenon.

When Nerolidol is encapsulated (LN), the picture becomes more complex. In Gram-positive strains, LN often retains the inhibitory capacity observed for free Nerolidol, and in some cases, inhibition appears at concentrations where the corresponding free NER already shows measurable effects. Moreover, since LN combines both lipid-related effects and Nerolidol activity, the interpretation necessarily requires comparison with UN at the same lipid level. When this comparison is made, it becomes evident that the presence of Nerolidol does not uniformly “enhance” antimicrobial action but rather modulates it in a strain- and concentration-dependent way.

Overall, the joint analysis of Figure 7 and Table 9 supports three key conclusions:

(i) antimicrobial responses are threshold-driven rather than linearly dose-dependent;
(ii) meaningful interpretation requires separating the contribution of the lipid matrix from that of Nerolidol; and
(iii) Nerolidol-loaded invasomes should be regarded as delivery platforms whose microbiological behavior depends on both components, rather than as inherently more potent antimicrobial agents. This perspective is essential for rationally designing future applications, particularly in contexts such as animal nutrition, where selectivity toward target microorganisms is critical.

## 4. Conclusions

In this work, we addressed the fabrication and characterization of liposomal systems containing Nerolidol, with particular emphasis on the relationship between loading level, vesicle structure, and colloidal stability within a manufacturing framework based on the simil-microfluidic coaxial injection technique. From a manufacturing perspective, the simil-microfluidic approach offers advantages in terms of continuous operation and process control. However, potential challenges may arise in scaling up the system, particularly in maintaining precise flow control, preventing fouling or clogging in continuous operation, and ensuring batch-to-batch reproducibility at larger production scales.

The investigation was deliberately developed in progressive stages. As a preliminary step, unloaded nanoliposomes were produced under different operating conditions, with the specific objective of “crystallizing” a reliable set of parameters able to guarantee reproducible vesicle formation. These experiments allowed the identification of process conditions that yielded liposomes characterized by Z-average values around 300 nm, acceptable polydispersity, and ζ-potential values indicative of good electrostatic stabilization. This step provided the technological baseline upon which subsequent loading trials could be rationally designed.

Starting from this reference system, Nerolidol was introduced in increasing amounts, moving from relatively high nominal loads down to lower values. This strategy clearly demonstrated that excessive Nerolidol incorporation progressively perturbs the structure of the phospholipid bilayer. High nominal loads were systematically associated with increased turbidity, vesicle enlargement, worsening of polydispersity, and signs of colloidal instability, consistent with excessive bilayer fluidization and loss of structural integrity. At the same time, encapsulation efficiency did not increase proportionally, indicating that simply forcing more Nerolidol into the ethanolic phase does not translate into higher effective payloads.

By progressively reducing the Nerolidol amount, a different scenario emerged. Formulations containing Nerolidol at approximately 1–2% of the lipid mass showed almost complete encapsulation, preserved vesicle morphology, Z-average values around 300 nm, and ζ-potential values exceeding the conventional stability threshold. These systems also exhibited more favorable stability over storage, suggesting that, within this loading window, Nerolidol can be accommodated in the bilayer without compromising its structural organization. The use of tangential flow filtration further contributed to obtaining purified suspensions while preserving vesicle integrity, confirming the compatibility of the process with downstream operations.

Taken together, these results indicate that the limiting factor in the development of Nerolidol-loaded invasomes is not the manufacturing approach itself, but rather the physicochemical compatibility between Nerolidol and the lipid bilayer. From a formulation standpoint, the realistic target is therefore not the maximization of the absolute loading but the identification of a balanced composition in which encapsulation efficiency, colloidal stability, and structural robustness coexist. The production process, including the sterile-oriented procedures adopted here, is compatible with applications requiring microbiological quality assurance. These findings provide external validation of the technological path followed in this study and strengthen its translational relevance.

An equally important dimension of the present work is the integration of microbiological evidence. The antimicrobial datasets, previously obtained on representative formulations, show that the biological response is threshold-driven and cannot be interpreted solely in terms of nominal lipid concentration. Free Nerolidol displayed activity mainly against Gram-positive bacteria, whereas Gram-negative species were poorly affected. Unloaded Nanoliposomes exhibited modest, concentration-dependent effects at the highest lipid levels, attributable to physical membrane–membrane interactions. Nerolidol-loaded invasomes combined both contributions: in several cases, they reproduced the activity of free Nerolidol, but did not systematically extend the antibacterial spectrum nor produce enhancements independent of lipid concentration. These findings emphasize that Nerolidol-loaded invasomes should be considered delivery platforms with dual behavior—where the active component and the carrier both contribute—rather than intrinsically more potent antimicrobials.

It is worth emphasizing that the novelty of this work lies in the identification of process–formulation constraints and loading limits for Nerolidol invasomes, rather than in the biological evaluation itself, which has been previously reported. The present study, therefore, provides a technological and mechanistic framework to guide future biological and translational investigations.

In conclusion, the present study demonstrates that the simil-microfluidic coaxial injection technique is suitable for the scalable preparation of Nerolidol-loaded invasomes, provided that the active loading remains within a well-defined window (≈1–2% *w*/*w*). The work clarifies the structural consequences of exceeding those limits and identifies a formulation range in which high encapsulation, acceptable polydispersity, and good stability can be simultaneously achieved. Within this framework, Nerolidol-loaded invasomes emerge not only as a scientifically interesting model system but also as a potentially versatile platform for future antimicrobial strategies, including potential applications of interest in the animal nutrition field, which should be considered as prospective and will require further investigation in terms of safety, dosage, and regulatory compliance. While Nerolidol is already used as a flavoring agent and feed additive, its incorporation into delivery systems such as invasomes requires additional evaluation from a regulatory and safety perspective. Further in vitro and in vivo studies will be required to translate these findings into practical scenarios.

## Figures and Tables

**Table 1:** Main properties of Nerolidol (BASF Safety Data Sheet).

Property	Value
Molecular Formula	C_15_H_26_O
State	Liquid
Molecular weight (g/mol)	222.37
CAS number	7212-44-4
Water solubility (mg/L) (20 °C)	14.1 * [BASF Safety Data Sheet]
Glass transition temperature (°C) (1 bar)	−90 [BASF Safety Data Sheet]
Boiling Point (°C) (1 bar)	276 [BASF Safety Data Sheet]
Log P	4.68 [BASF Safety Data Sheet]
Solubility	high in ethanol–low in water
Topological surface area (Å)	20.23
Van der Waals Molecular volume (Å^3^/molecule)	268.93

* 1.532 (25 °C) Experimental (https://hmdb.ca/metabolites/HMDB0035662, accessed on February 2026).

**Table 2:** Examples of Nerolidol used as a penetration enhancer.

Used Delivery System	NER Enhancer to Active Ingredient	References
Hydrogel	Curcumin	[6]
Hydrogel	Hydrocortisone Hydrochloride Carbamazepine Tamoxifen	[18,31]
Hydrogel	Selegiline hydrochloride	[18]
Hydrogel	Terbinafine	[30]
Chitosan gel	Ondansetron hydrochloride	[30]
Invasome	Buprenorphine hydrochloride Bupivacaine	[28]
Hydrogel	Hydrocortisone	[30]
Hydrogel	Propanolol hydrochloride	[30,32]
PG gel	Lomerizine dihydrochloride	[30]

**Table 3:** Theoretical concentrations in Nerolidol of prepared batches.

Batch	NER, (g)	Theoretical Load, %
Prod. 1 (UN)	0	0
INV. NER, 1%	0.0285	1
INV. NER, 5%	0.1480	5
INV. NER, 10%	0.3133	10
INV. NER, 15%	0.4980	15

**Table 4:** Batches at low theoretical concentrations of Nerolidol.

Batch	NER, (g)	Theoretical Load, %
INV. NER, 1%	0.0285	1
INV. NER, 2%	0.0576	2

**Table 5:** Dimensional and turbidimetric characterization of different batches of invasomes produced with varying loading.

Produced Batch	Z-Average [nm]	PDI	Turbidity [NTU]
Prod. 1 (UN)	305.08 ± 24.2	0.33 ± 0.03	318
INV. NER, 1%	309.99 ± 9.0	0.34 ± 0.00	238
INV. NER, 5%	604.00 ± 55.2	0.6 ± 0.07	589
INV. NER, 10%	697.00 ± 32.4	0.62 ± 0.04	912
INV. NER, 15%	981.00 ± 196.6	0.59 ± 0.04	1238

**Table 6:** Effective load and encapsulation efficiency of invasomes batches.

Produced Batch	Effective Load [%]	Efficiency [%]
INV. NER, 1%	1.00 ± 0.01	99.70 ± 0.42
INV. NER, 5%	4.93 ± 0.02	99.09 ± 1.00
INV. NER, 10%	9.92 ± 0.08	99.27 ± 0.90
INV. NER, 15%	14.98 ± 0.007	99.76 ± 0.27

**Table 7:** Dimensional characterization of the four different production batches with variable loading immediately after production and after 6 months of storage (asterisked data refers to long-term stability).

Produced Batch	Z-Average [nm]	PDI	Z-Average [nm] *	PDI *
INV. NER, 1%	309.99 ± 9.03	0.34 ± 0.00	329.6 ± 8.99	0.69 ± 0.13
INV. NER, 5%	604.10 ± 55.0	0.60 ± 0.07	467.7 ± 6.58	0.59 ± 0.07
INV. NER, 10%	697.05 ± 32.5	0.62 ± 0.04	372.0 ± 20.55	0.87 ± 0.03
INV. NER, 15%	981.00 ± 196.6	0.59 ± 0.04	477.9 ± 75.56	0.84 ± 0.05

**Table 8:** Size, Z-potential, effective loading, and encapsulation efficiency for invasomes immediately after the production and after 45 days of storage.

Produced Batch *	Z-Average, [nm]	PDI	Z-Potential, [mV]	Effective Load, [%]	Efficiency [%]
INV. NER, 1%	298.8 ± 9.84	0.489 ± 0.097	−42.36 ± 5.4	1.190	100
INV. NER, 2%	301.8 ± 19.99	0.605 ± 0.106	−52.18 ± 6.4	2.02	99.34
INV. NER, 1% Aged	341.7 ± 9.98	0.64 ± 0.04	−49.81 ± 0.8	1.08 ± 0.05	90.7
INV. NER, 2% Aged	342.8 ± 8.22	0.60 ± 0.11	−51.41 ± 0.89	1.44 ± 0.11	71.1

* Since for both the loading ratio the *p*-values are larger than 0.05, no statistically significant differences were observed due to the aging process.

**Table 9:** Qualitative interpretation of antimicrobial responses for each bacterial strain exposed to free Nerolidol (NER), Unloaded Nanoliposomes (UN) and Nerolidol-loaded invasomes (LN). The table synthesizes whether inhibition is present and whether it displays a dose-dependent pattern, based on the re-evaluation of the Supplementary time–kill data from [33].

Strain	ATCC	Gram	Killing Effects		
			LN	UN	Free NER
*Staphylococcus aureus* (MRSA)	43,300	(+)	Yes, dose dependent	Yes, dose dependent	Yes
*Enterococcus faecium*	19,434	(+)	Yes, slightly dose dependent	Yes, slightly dose dependent	Yes
*Lactobacillus acidophilus*	4356	(+)	Yes, dose independent	Yes, dose independent	Yes
*Salmonella typhimurium*	14,028	(−)	Yes, slightly dose dependent	Not for all concentrations	No
*Escherichia coli*	25,922	(−)	Yes, dose dependent	Not for all concentrations	No

## Data Availability

Data sharing is not applicable to this article.
